# Health state utilities for beta-thalassemia: a time trade-off study

**DOI:** 10.1007/s10198-022-01449-7

**Published:** 2022-03-26

**Authors:** Antony P. Martin, Enrico Ferri Grazzi, Claudia Mighiu, Manoj Chevli, Farrukh Shah, Louise Maher, Anum Shaikh, Aliah Sagar, Hayley Hubberstey, Bethany Franks, Juan M. Ramos-Goñi, Mark Oppe, Derek Tang

**Affiliations:** 1 Formerly HCD Economics, Daresbury, UK; 2 HCD Economics, Daresbury, UK; 3 Celgene Ltd, a Bristol-Myers Squibb Company, Uxbridge, UK; 4 University College London Hospital, London, UK; 5 Formerly Axentiva Solutions, Tacoronte, Santa Cruz de Tenerife, Spain; 6 Bristol Myers Squibb, Princeton, NJ USA

**Keywords:** Beta-thalassemia, Hemoglobin, Anemia, Time trade-off, Transfusion burden, I12 Health Behavior

## Abstract

**Background:**

Beta-thalassemia (BT) is an inherited blood disorder characterized by reduced levels of functional hemoglobin resulting in phenotypes ranging from clinically asymptomatic to severely anemic. Patients with BT may require lifelong regular blood transfusions supported by appropriate iron chelation therapy (ICT). This study aimed to determine how the UK general population values BT health states associated with differing transfusion burden and ICT.

**Methods:**

Composite time trade-off (cTTO) methodology was employed to elicit health state utilities in BT. Relevant BT literature related to symptom and quality-of-life impact, including physical, functional, and emotional well-being, and safety profiles of BT treatments were considered when drafting health state descriptions. Eleven health state descriptions were developed and validated by hematologists and patient advocates for clinical accuracy and completeness. 200 individuals from the UK general population participated in the cTTO interviews.

**Results:**

The mean age of participants was 41.50 years (SD 16.01, range 18–81); 88 (46.8%) were female. Utility values ranged from 0.78 (SD 0.34) for non-transfusion dependent BT with oral ICT to 0.37 (SD 0.50) for high transfusion burden with subcutaneous ICT in transfusion-dependent BT.

**Conclusions:**

This study provides health utilities for a range of BT health states from the UK general population perspective. Importantly, lower transfusion burden and lower burden of anemia were associated with higher utilities. To a lesser extent, differential modes of ICT were found to impact utility valuations in patients with BT. The utilities obtained in this study can be employed as inputs in cost-effectiveness analyses of BT therapies.

**Supplementary Information:**

The online version contains supplementary material available at 10.1007/s10198-022-01449-7.

## Introduction

Beta-thalassemia (BT) comprises a group of inherited blood disorders characterized by reduced or absent functional hemoglobin (Hb) in red blood cells. The different phenotypes (major, intermediate, and minor) cause a range of symptoms. BT may cause weakness, fatigue, and serious complications such as an enlarged liver or spleen and increased risk of venous thrombosis [1, 2]. Patients with transfusion-dependent BT (TDT) require regular blood transfusions to maintain Hb levels, supported by iron chelation therapy (ICT) to control excess iron levels resulting from regular transfusions. Patients with non-TDT (NTDT) do not require regular transfusions; however, symptoms vary from mild clinical problems to severe complications, such as skeletal problems and growth retardation [3]. Both types require frequent hospital visits and blood tests, which may affect daily life [4, 5].

BT is rare, with approximately 1200 patients registered in England in 2019, according to the National Haemoglobinopathy Registry [6, 7]. As new treatments with the potential to reduce transfusion burden and associated ICT are developed, more information is needed about the relationship between this burden and health-related quality of life (HRQoL) [8, 9]. Quality of life (QoL) benefits derived from treatment approaches may be quantified using health state valuations (HSVs). The resultant utility values are defined by the strength of an individual’s preference for being in a particular health state, quantified into an index value on a scale from 0 (death) to 1 (full health) [10].

Recently, Matza et al. estimated HSVs associated with hematopoietic stem cell transplantation, as a one-off alternative to a lifelong transfusion schedule [11]. Although potentially providing a cure, this treatment approach is associated with high risk of complications, such as transplant-related toxicity and graft-versus-host disease [11–13]. Nonetheless, it remains a clinical option for the management of BT in children and young adults in the UK, although compatibility issues may compromise treatment eligibility [6, 14–18]. Other studies have examined associated HSVs with transfusions and ICT in diseases such as myelodysplastic syndromes [19–22].

In the absence of published EuroQol 5-dimension (EQ-5D) utilities for transfusion-related BT health states, either directly observed or obtained from mapping, a time trade-off (TTO) study was proposed to obtain HSVs. To date, the paucity of published utilities may be due to reduced sensitivity of the EQ-5D instrument and other generic measures to disease-specific treatment process attributes [11, 23–25] and the rarity of BT [6, 26, 27]. The TTO approach offers a simple method for respondents to relate preferences to time, resulting in lower cognitive burden compared to risk-based methods, such as the standard gamble. Furthermore, TTO is the preferred choice-based method according to UK National Institute for Health and Care Excellence (NICE) guidelines, indicating a preference toward valuation methods similar to those used in the 3-level EQ-5D (EQ-5D-3L) valuation study, which followed the TTO measurement and valuation of health (MVH) protocol [26, 28–31]. Such a protocol, however, prescribes fundamentally different methods for the valuation of better-than-dead and worse-than-dead health states, as opposed to the newly developed EuroQol Valuation Technology (EQ-VT) protocol, which uses the lead-time TTO (LT-TTO) methodology for the valuation of worse-than-dead health states [31–33].

The aim of this study was to elicit robust HSVs corresponding to health states for BT, using a TTO valuation approach. The primary objective was to quantify the utility impact of BT overall (not only associated with transfusion burden) from a societal perspective. Secondary objectives were to explore how HSVs differed by transfusion burden and ICT modality.

## Methods

### Overview of composite TTO (cTTO) methodology

The cTTO methodology was implemented to derive HSVs. The cTTO protocol was developed by the EuroQol Group in EQ-VT protocol version 2.1 for 5-level EQ-5D (EQ-5D-5L) valuation studies and was adopted for this research [31, 33, 34]. This protocol is considered best practice for conducting cTTO studies and has addressed several limitations of earlier TTO approaches. As of version 1.1, the EQ-VT protocol is paired with a quality control (QC) procedure to review protocol compliance and interviewer effects during data collection [35].

The cTTO task was completed in a computer-assisted personal interview. Incoming data were monitored to provide the interviewers with feedback on their performance. The interview tool was built in Microsoft PowerPoint, adapted from EuroQol’s Portable Valuation Technology. The tool stored the TTO values alongside paradata required for QC [35]. For each health state, respondents were asked if they would prefer to live in the health state described for 10 years, followed by death, or whether they would rather have a life in full health for a shorter duration, *t*. The value for *t* was iteratively varied until the point of indifference was found, where the respondent was unable to choose [31].

If respondents preferred immediate death to living in a health state for 10 years, the cTTO task was completed using the LT-TTO [32, 36]. This involved choosing between a short life of *t* years in full health (starting duration of 10 years) or a longer life with 10 years in full health followed by 10 years in the health state (a combined duration of 20 years), with *t* varied to find the point of indifference. This method allowed the elicitation of negative utility values. In summary, the cTTO task used the conventional TTO approach for the evaluation of better-than-dead states, and the LT-TTO approach for valuation of worse-than-dead states [31, 34].

### Development of health state descriptions

The health state descriptions were developed to ensure clinical relevance, clarity, and comprehensibility for study participants. A targeted literature review was conducted to identify patient-reported outcome measures (PROMs) and QoL assessment in BT. Health state descriptions were developed based on symptoms and daily burden of BT, as well as the existing treatment paradigm; they were further informed by previous studies, identified through a targeted literature review, in terms of aspects of physical, functional, and emotional well-being, and adverse events [6, 11–22].

Draft health state descriptions were designed and reviewed with input from three expert physicians specialized in hemoglobinopathies with experience in treating BT, and two patient advocates, including one patient and one caregiver/advocate.

**Fig. 1:**
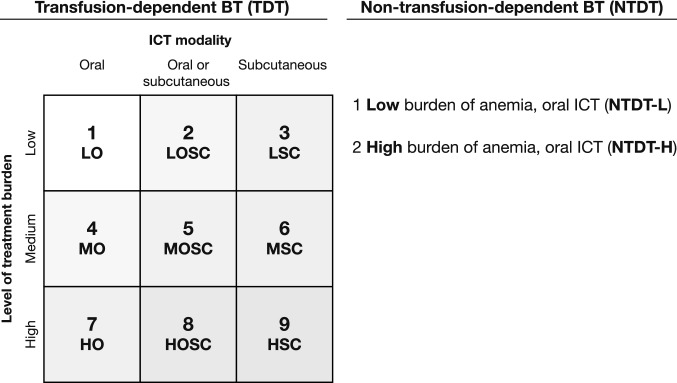
Final health states for TDT and NTDT. *HO* high burden (O-ICT), *HOSC* high burden (O- and SC-ICT), *HSC* high burden (SC-ICT), *ICT* iron chelation therapy, *LO* low burden (O-ICT), *LOSC* low burden (O- and SC-ICT), *LSC* low burden (SC-ICT), *MO* medium burden (O-ICT), *MOSC* medium burden (O- and SC-ICT), *MSC* medium burden (SC-ICT), *NTDT* non-TDT, *NTDT-H* non-TDT (high burden of anemia), *NTDT-L* non-TDT (low burden of anemia), *O-ICT* oral ICT, *SC-ICT* subcutaneous ICT, *SD* standard deviation, *TDT* transfusion-dependent beta-thalassemia, *MRI* magnetic resonance imaging

To ensure clinical accuracy and completeness, the team drafted the health state descriptions based on the results of the targeted literature review and iteratively revised the descriptions based on feedback from the expert physicians following a pilot study. The experts were provided with the vignettes and asked to provide feedback according to a semi-structured interview methodology [37, 38]. Example vignettes are available in the Online Resource Appendix. The final health state descriptions were developed, including nine related to TDT health states based on level of treatment burden (low [every 4–5 weeks]; medium [every 4 weeks]; high [every 3 weeks]) and type of ICT: (1) low transfusion burden with oral ICT (O-ICT) (LO), (2) low transfusion burden with O-ICT or subcutaneous ICT (SC-ICT) (LOSC), (3) low transfusion burden with SC-ICT (LSC), (4) medium transfusion burden with O-ICT (MO), (5) medium transfusion burden with O-ICT or SC-ICT (MOSC), (6) medium transfusion burden with SC-ICT (MSC), (7) high transfusion burden with O-ICT (HO), (8) high transfusion burden with O-ICT or SC-ICT (HOSC), (9) high transfusion burden with SC-ICT (HSC). Two health states for NTDT based on low versus high burden of anemia (Fig. 1) were developed: (1) NTDT with O-ICT (low burden of anemia) (NTDT-L), and (2) NTDT with O-ICT (high burden of anemia) (NTDT-H).

### Validation of health state descriptions and pilot interviews

The participating BT patient and caregiver were involved in validating the health states. Following the first round of validation and refinement of the health state descriptions, to examine feasibility, a pilot study was undertaken with a small sample of the general public (*n* = 5; 80% male; mean age 49.6 years). Participants reviewed all health states and valued them with the cTTO task. Participants were asked a series of questions to ensure that the description wording was well understood; all participants reported that the language and content were clear and coherent. Minor formatting edits were suggested, and the descriptions were amended accordingly. Following the pilot interviews, additional feedback was obtained from one of the patient advocates and a third independent clinical expert to further refine the descriptions.

### Participants

In accordance with the MVH protocol, to quantify the impact of BT from a societal perspective, members of the UK general population were invited to participate in the study [39]. The research team aimed to match the participant sample with the general population by age and gender [40, 41]. A sample size of 200 was chosen based on observations from the published literature, and the EuroQol group, which recommends a minimum of 100 observations per health state [42].

Participants were enrolled through online advertising, flyers, and word of mouth via two recruiters. Minimum recruitment quotas were applied based on age and gender with the aim to achieve a sample with a reasonable degree of similarity to the UK general population (based on Office for National Statistics [ONS] data for August 2019) [41]. Reasonable compensation (a GBP 30 Amazon/high street voucher) was provided as reimbursement for time and travel.

A sample size of at least 200 was targeted, based on observations from the published literature and the EuroQol group recommendation of a minimum of 100 responses per health state [11, 42, 43].

### Interview setting and procedure

A total of 332 participants were scheduled for interviews: 205 attended the scheduled interview, and 200 completed the cTTO questionnaire. Individual interviews were carried out face to face in Manchester, UK, by five trained interviewers using the EQ-VT. All participants were provided with written and verbal descriptions of the exercise, as well as instructions on completing the cTTO questionnaire. Participants provided informed consent, responded to demographic questions, were guided through example questions, and completed practice tasks before evaluating the BT health states. A disease information sheet, detailing basic information on BT was provided to respondents to familiarize themselves with the condition before initiating the BT tasks. Health states were presented to the participants one by one in random order. After the survey, participants had the opportunity to flag any erroneous valuations through the feedback module of the EQ-VT, which reported the ranking of vignettes according to their valuations to ensure responses reflected their preferences [44, 45].

### QC and outcomes

It is recommended that QC is continuous throughout the TTO process to ensure an optimal environment for data collection [35]. QC parameters collected and analyzed for each interview included: (a) time to complete the example task (the “wheelchair” example), where at least 3 min was recommended; (b) time to complete the TTO HSV tasks, where at least half a minute per health state was recommended; and (c) the number of interviews in which the LT-TTO task was not shown to the participant in the “wheelchair” example.

For each interviewer, the number of interviews with non-traders (i.e., people who chose the HSV of 1 for all health states) and the number of interviews with non-negative HSVs were recorded. The following markers for data quality were assessed: (a) interviews with at least one worse-than-dead HSV; (b) interviews without a worse-than-dead value, but 0 utility as the bottom value for one or more health states; and (c) interviews with worse-than-dead values where the participant assigned a HSV of 1 for one or more health states. Interviewer effects were assessed to identify any possible bias in data collection, including: (a) number of worse-than-dead values; (b) number of non-traders; (c) number of zero values; (d) average number of moves per health state; and (e) average HSVs. Face validity of the data was assessed, to determine whether elicitations were in accordance with a logical differentiation of values following severity levels and if gaps were present in the distributions. Distributions were calculated and compared across health states and across interviewers to identify any major deviations between interviewers.

The primary study outcome was a HSV for each health state description. As such, it was considered appropriate to incorporate participants only if they understood the assignment. To do so, exclusion criteria were applied in line with common practice [46]. With reference to the EQ-5D-5L UK value set development, it was deemed that responses should be excluded if: (a) a participant gave the same HSV to all health states; or (b) a participant chose a HSV of 1 for all health states (non-trading) [29]. However, data from participants valuing the worst health states no lower than the mildest state were not excluded, as perception of BT health state severity is considered more proximate than possible combinations of the EQ-5D-5L.

### Statistical analysis

Descriptive statistics were used to describe HSVs and key characteristics of the sample. Paired *t*-tests were performed to test for differences between pairs of health states. Analysis of variance (ANOVA) and post hoc analyses with Bonferroni correction were conducted to compare utilities between age and gender subgroups. Non-parametric analyses, using the Kruskal–Wallis test with post hoc analysis with Dunn’s test, were performed to ensure accuracy of results. Two scenario analyses (one including non-trading results and one excluding observations flagged by respondents) were also performed to assess the robustness of results. All analyses were performed using Stata Statistical Software: Release 16, 2019 (StataCorp, College Station, TX, USA).

## Results

### Main attributes of survey participants

The following sociodemographic characteristics were recorded for each participant: age, sex, ethnicity, marital status, employment status, and education level.

Of 200 participants who completed the cTTO questionnaire, 12 were excluded for non-trading (valuing all health states as 1), or for giving the same HSV to all health states. Of the remaining 188 participants, 46.8% were female, mean age was 41.5 years (standard deviation [SD] 16.01; range 18–81), 74.5% were white, and most were employed (full-time 44.1%; part time 21.8%; Table 1). The overall demographic distribution of the study sample was similar to the general population [47–50].

**Table 1:** Demographic characteristics

	Sample (*n* = 188)	UK general population
Age, mean (SD), years^a^ [40, 41]	41.5 (16.01)	40.2
Age categories, *n* (%)^a^ [40, 41]		
18–35	80 (42.6)	30.8
36–55	59 (31.4)	32.0
≥56	49 (26.1)	37.2
Sex, *n* (%) [40]		
Male	96 (51.0)	49.4
Female	88 (46.8)	50.6
Other	4 (2.1)	–
Ethnicity, *n* (%) [47]		
White	140 (74.5)	86.0
Mixed/multiple ethnic groups	12 (6.4)	2.2
Asian or Asian British	11 (5.9)	7.5
Black/African/Caribbean/Black British	20 (10.6)	3.3
Other ethnic groups	4 (2.1)	1.0
Prefer not to say	1 (0.5)	N/A
Marital status, *n* (%) [48]		
Single	102 (54.3)	35.0
Married/domestic partner	65 (34.6)	50.6
Separated/divorced	11 (5.9)	8.0
Widowed	2 (1.1)	6.4
Other	7 (3.7)	–
Prefer not to say	1 (0.5)	–
Employment status, *n* (%)^b^ [49]		
Full-time employed^c^	83 (44.1)	46.6
Part-time employed^c^	41 (21.8)	16.3
Unemployed^d^	50 (26.6)	16.5
Retired	14 (7.4)	20.6
Education, *n* (%)^e^ [50]		
Postgraduate level^f^	25 (13.3)	42.0
University level	66 (35.1)	
A-levels or equivalent	48 (25.5)	21.0
GCSEs or equivalent	32 (17.0)	20.0
None of the above	17 (9.0)	8.0

*GCSE* General Certificate of Secondary Education, *N/A* not applicable, *SD* standard deviation

^a^All statistics regarding the general population reported above exclude population aged younger than 16 years

^b^Employment status categories are reported as an absolute percentage of the general population to allow for comparison

^c^Part-time and full-time also include self-employed

^d^Unemployed also includes economically inactive (for any reason) 16–64 years of age

^e^The education level distribution is based on data for people between the ages of 20 and 64 years who are not in education

^f^Postgraduate level and university level are considered in the same category by the Office for National Statistics

### Health state utilities

The main task entailed the valuation of all 11 health states in random order. For the non-transfusion dependent health states, respondents gave a higher mean utility to the low anemia burden health state, NTDT-L (0.78; SD 0.34) than the high anemia burden health state, NTDT-H (0.63; SD 0.40). For the transfusion-dependent health states, LOSC had the highest mean utility (0.75; SD 0.30), and HSC had the lowest (0.37; SD 0.50). The rank and mean of the health states (Table 2) were ordered as expected.

**Table 2:** Mean health state utility values (*n* = 188)

HSV^a^	Mean (SD)	Median
NTDT population		
NTDT-L	0.78 (0.34)	0.9
NTDT-H	0.63 (0.40)	0.75
TDT population		
LOSC	0.75 (0.30)	0.85
LO	0.74 (0.33)	0.85
LSC	0.70 (0.36)	0.8
MO	0.60 (0.42)	0.7
MOSC	0.57 (0.39)	0.7
MSC	0.54 (0.47)	0.7
HO	0.43 (0.50)	0.6
HOSC	0.37 (0.49)	0.5
HSC	0.37 (0.50)	0.5

*HO* high burden (O-ICT), *HOSC* high burden (O- and SC-ICT), *HSC* high burden (SC-ICT), *HSV* health state valuation, *LO* low burden (O-ICT), *LOSC* low burden (O- and SC-ICT), *LSC* low burden (SC-ICT), *MO* medium burden (O-ICT), *MOSC* medium burden (O- and SC-ICT), *MSC* medium burden (SC-ICT), *NTDT* non-TDT, *NTDT-H* non-TDT (high burden of anemia), *NTDT-L* non-TDT (low burden of anemia), *O-ICT* oral iron chelation therapy, *SC-ICT* subcutaneous ICT, *SD* standard deviation, *TDT* transfusion-dependent beta-thalassemia

^a^HSVs are presented from highest to lowest value

### Utility differences

*T-*tests confirmed that the differences between transfusion-dependent HSVs were significant at the *p* < 0.00001 level between transfusion burden levels (Table 3). However, mean differences for transfusion-dependent HSVs were not all statistically significant at the *p* < 0.05 level for health states with differing ICT modalities within the same transfusion burden level (Table 4). The observed differences and the significance levels suggested that as transfusion burden decreased, the O-ICT strategy remained the preferred option; however, in the low transfusion burden group (LO, LSC, LOSC), the O-ICT strategy was not the preferred option. This result may be related to a smaller impact of ICT modality compared to transfusion frequency and impact on daily life, which becomes more apparent in lower transfusion burden health states—i.e., respondents may have reacted more strongly to frequency of transfusion and impact on daily life than to method of ICT drug administration.

**Table 3:** *T*-tests of mean difference: TDT health states between transfusion burden levels

HSV^a^	Mean difference	SD	95% CI	*p* value
SC-ICT				
LSC–MSC*	0.16	0.41	0.10–0.22	<0.00001
LSC–HSC*	0.33	0.47	0.26–0.40	<0.00001
MSC–HSC*	0.17	0.47	0.10–0.24	<0.00001
O-ICT				
LO–MO*	0.13	0.35	0.08–0.18	<0.00001
LO–HO*	0.31	0.46	0.24–0.38	<0.00001
MO–HO*	0.17	0.39	0.11–0.23	<0.00001
O- or SC-ICT				
LOSC–MOSC*	0.18	0.36	0.13–0.23	<0.00001
LOSC–HOSC*	0.38	0.45	0.32–0.44	<0.00001
MOSC–HOSC*	0.20	0.38	0.14–0.25	<0.00001

*CI* confidence interval, *HO* high burden (O-ICT), *HOSC* high burden (O- and SC-ICT), *HSC* high burden (SC-ICT), *HSV* health state valuation, *ICT* iron chelation therapy, *LO* low burden (O-ICT), *LOSC* low burden (O- and SC-ICT), *LSC* low burden (SC-ICT), *MO* medium burden (O-ICT), *MOSC* medium burden (O- and SC-ICT), *MSC* medium burden (SC-ICT), *O-ICT* oral ICT, *SC-ICT* subcutaneous ICT, *SD* standard deviation, *TDT* transfusion-dependent beta-thalassemia

^a^The differences are grouped according to ICT strategy

^*^Statistically significant (*p* < 0.05)

**Table 4:** *T*-tests of mean differences: TDT health states within transfusion burden levels (*n* = 188)

HSV^a^	Mean difference	SD	95% CI	*p* value
High burden				
HO–HSC*	0.06	0.41	0.00–0.12	0.03
HO–HOSC*	0.06	0.38	0.01–0.12	0.02
HOSC–HSC	0.00	0.36	− 0.05 to 0.05	0.98
Medium burden				
MO–MSC*	0.07	0.43	0.00–0.13	0.03
MO–MOSC	0.04	0.38	0.02–0.09	0.20
MOSC–MSC	0.03	0.43	− 0.03 to 0.09	0.32
Low burden				
LO–LSC*	0.04	0.27	0.00–0.08	0.03
LO–LOSC	− 0.01	0.25	− 0.05 to 0.03	0.56
LOSC-LSC	0.06	0.41	0.02–0.09	0.01

*CI* confidence interval, *HO* high burden (O-ICT), *HOSC* high burden (O- and SC-ICT), *HSC* high burden (SC-ICT), *HSV* health state valuation, *ICT* iron chelation therapy*, LO* low burden (O-ICT), *LOSC* low burden (O- and SC-ICT), *LSC* low burden (SC-ICT), *MO* medium burden (O-ICT), *MOSC* medium burden (O- and SC-ICT), *MSC* medium burden (SC-ICT), *O-ICT* oral ICT, *SC-ICT* subcutaneous ICT, *SD* standard deviation, TDT transfusion-dependent beta-thalassemia

^a^The differences are grouped according to transfusion burden

^*^Statistically significant (*p* < 0.05)

NTDT-L HSVs were compared with TDT low-burden health states, as their utilities held the closest values and were deemed most relevant for comparison. For diligence, the NTDT-H valuations were still compared with both transfusion-dependent low and medium burden, but the medium burden was considered the most relevant for comparison (Table 5). The difference between NTDT-L and low transfusion burden health states showed *p* values below the 0.05 threshold only when compared with LSC (*p* = 0.0008), suggesting that NTDT-L, while preferred by most participants, had a HSV relatively similar to LO. Differences between NTDT-H and low and medium burden health states yielded *p* values below the *p* < 0.05 level in all cases apart from MO (Table 5). The differences indicated that lower transfusion burden was associated with higher utility; however, for the NTDT-H health state, no significant difference from MO was found.

**Table 5:** *T*-tests of mean differences: NTDT health states and within TDT health states transfusion burden levels (*n* = 188)

HSV^a^	Mean difference	SD	95% CI	*p* value
Non-transfusion dependent (low burden)–low transfusion burden				
NTDT-L–LO	0.04	0.32	0.01–0.08	0.12
NTDT-L–LSC*	0.08	0.33	0.03–0.13	0.0008
NTDT-L–LOSC	0.03	0.34	0.02–0.08	0.25
Non-transfusion dependent (high burden)–low transfusion burden				
NTDT-H–LO*	− 0.11	0.35	− 0.16 to 0.05	0.0001
NTDT-H–LSC*	− 0.06	0.40	− 0.12 to 0.00	0.0326
NTDT-H–LOSC*	− 0.12	0.39	− 0.18 to 0.06	0.0001
Non-transfusion dependent (high burden)–medium transfusion burden				
NTDT-H–MO	0.03	0.41	− 0.03 to 0.09	0.31
NTDT-H–MSC*	0.10	0.49	0.03–0.17	0.007
NTDT-H–MOSC*	0.07	0.42	0.01–0.13	0.031

*CI* confidence interval, *HSV* health state valuation, *ICT* iron chelation therapy, *LO* low burden (O-ICT), *LOSC* low burden (O- and SC-ICT), *LSC* low burden (SC-ICT), *MO* medium burden (O-ICT), *MOSC* medium burden (O- and SC-ICT), *MSC* medium burden (SC-ICT), *NTDT* non-TDT, *NTDT-H* non-TDT (high burden of anemia), *NTDT-L* non-TDT (low burden of anemia), *O-ICT* oral ICT, *SC-ICT* subcutaneous ICT, *SD* standard deviation, *TDT* transfusion-dependent beta-thalassemia

^a^The differences are grouped according to NTDT burden and transfusion burden

^*^Statistically significant (*p* < 0.05)

The sample was divided into age groups with the aim of performing subgroup analysis to ascertain whether inter-generational differences existed in HSVs. The age groups were <35, 36–55, and >55 years, representing 42.5% (*n* = 80), 31.4% (*n* = 59), and 26.1% (*n* = 49) of our sample, respectively. Selection of the age groups was conducted with the objective of achieving a distribution similar to that of the three generations within the adult UK general population, with the age groups representing 30.8, 32.0 and 37.2% of the population > 16 years of age, respectively. Additional subgroup analysis was performed with respect to gender.

No significant differences in HSV by gender were observed when ANOVA with Bonferroni correction was performed. For eight health states, non-parametric analysis (Kruskal–Wallis and Dunn’s test) performed after ANOVA confirmed no significant differences between age groups. However, lower HSVs (*p* < 0.05) were associated with the >55 years age group when compared to the <35 and 35–55 years age groups for LOSC (0.63 vs 0.82 vs 0.77), MSC (0.37 vs 0.63 vs 0.59), and HO (0.23 vs 0.54 vs 0.52), respectively.

### Quality of interviews

#### Protocol compliance

For the included sample (*n* = 188), the average time to completion was 32.6 min (SD 10.1). On average, the “wheelchair” examples and BT-specific tasks lasted 6.9 (SD 2.8) and 21.9 min (SD 7.6), respectively. Nine interviews had a “wheelchair” example duration of less than 3 min; however, in three cases this was due to PowerPoint software failure, therefore only six were deemed non-compliant. Although 17 interviews underwent quality assessment to ensure consistency in data collection, no issues relating to data quality were identified. No interviews were reported to have a duration of less than 5.5 min for the BT-specific tasks.

Twenty-two interviews were identified for data review based on the pattern of responses. Review of the interviews indicated that there were no quality issues, and the data were included in the sample. The number of interviews with at least one worse-than-dead HSV was 41 (21.8%) in the BT tasks. A total of 70 interviews (37.2%) had at least one health state valued at 1, and 35 (18.6%) had no worse-than-dead HSV but at least one valued at 0.

#### Interviewer effects

All interviewers had at least one non-trader, apart from Interviewer 5 (Online Resource Fig. S1), who had both a higher number of interviews (Table 6) and a higher number of non-traders. The density of zero values was found to be relatively consistent across interviewers and to be left-skewed (Online Resource Fig. S2). In addition, the distribution of worse-than-dead values across interviewers was assessed and found to be similar and characterized by a marked left skewness, as most participants considered the health states better than death (Online Resource Fig. S3). The similarity of worse-than-dead value distributions across interviewers implied the absence of substantial interviewer impact on the elicited values.

**Table 6:** Overall number of interviews and number of flagged interviews per interviewer

Interviewer	Total interviews		Flagged interviews	
	Frequency	Percentage	Frequency	Percentage^a^
Interviewer 1	24	12.00	0	0
Interviewer 2	51	25.50	3	5.88
Interviewer 3	32	16.00	6	18.75
Interviewer 4	26	13.00	4	15.38
Interviewer 5	67	33.50	9	13.43
Total	200	100.00	22	–

^a^Percentage of the interviews carried out by each interviewer that were flagged for any reason

The pattern for the average number of moves per interview was consistent across interviewers with a mean of 5.25 moves (SD 2.63), except for Interviewer 4, who had a much wider spread (Online Resource Fig. S4). The mean HSV was found to be 0.60, with only values from Interviewer 2 and 3 deviating appreciably from the mean (0.64 and 0.57, respectively). The median HSV was 0.68 with only values from Interviewer 2 deviating considerably (median 0.73; Online Resource Fig. S5).

#### Face validity of data

No notable gaps were observed, and data distributions followed a consistent logical pattern, in line with the severity of the different health states. However, greater variability was found in more severe health states, particularly HSC, HOSC, HO, and MSC. Distributions across interviewers for these health states had a similar shape, suggesting greater disagreement among respondents for health states further away from full health. Details available in Online Resource Fig. S6 to Fig. S17.

#### Scenario analyses

A scenario analysis was conducted to assess the potential effects that including the 12 non-traders may have had on the final HSVs elicitation. After their inclusion in the sample (*n* = 200), greater variability was observed in the HSVs across health states. Variability in HSVs was lower for milder states and greater for more severe states. This is due to the inclusion of non-traders, who, with a constant valuation of 1, increased the mean HSV. A second scenario analysis excluding the observations highlighted by the respondents in the feedback module showed little variation in both mild and severe states (Table 7). The ranking of the health states was affected only in the second scenario, where the value for HSC was slightly higher than HOSC; however, the scenario analyses overall demonstrated the robustness of the utility data elicited in this study.

**Table 7:** Comparison of mean HSVs

HSV^a^	Base case (*n* = 188)	Scenario 1 (*n* = 200)	Scenario 2 (feedback)
	Mean (SD)	Mean (SD)	Mean (SD), *n*
NTDT-L	0.78 (0.34)	0.78 (0.36)	0.78 (0.32), 179
LOSC	0.75 (0.30)	0.75 (0.32)	0.75 (0.31), 176
LO	0.74 (0.33)	0.74 (0.35)	0.75 (0.33), 167
LSC	0.70 (0.36)	0.70 (0.37)	0.69 (0.36), 175
NTDT-H	0.63 (0.40)	0.65 (0.41)	0.65 (0.40), 165
MO	0.60 (0.42)	0.62 (0.43)	0.61 (0.41), 177
MOSC	0.57 (0.39)	0.58 (0.42)	0.57 (0.41), 174
MSC	0.54 (0.47)	0.55 (0.48)	0.52 (0.48), 178
HO	0.43 (0.50)	0.46 (0.52)	0.42 (0.51), 178
HOSC	0.37 (0.49)	0.40 (0.50)	0.35 (0.49), 174
HSC	0.37 (0.50)	0.40 (0.52)	0.36 (0.50), 176

*HO* high burden (O-ICT), *HOSC* high burden (O- and SC-ICT), *HSC* high burden (SC-ICT), *HSV* health state valuation, *ICT* iron chelation therapy, *LO* low burden (O-ICT), *LOSC* low burden (O- and SC-ICT), *LSC* low burden (SC-ICT), *MO* medium burden (O-ICT), *MOSC* medium burden (O- and SC-ICT), *MSC* medium burden (SC-ICT), *NTDT* non-TDT, *NTDT-H* non-TDT (high burden of anemia), *NTDT-L* non-TDT (low burden of anemia), *O-ICT* oral ICT, *SC-ICT* subcutaneous ICT, *SD* standard deviation, *TDT* transfusion-dependent beta-thalassemia

^a^HSVs are presented from highest to lowest value

## Discussion

This study used the cTTO method to evaluate individual preferences for BT-related health states in a sample of the UK general population. The process of health state development included semi-structured interviews with BT clinical experts and patient advocates, allowing health state descriptions to capture important elements of the clinical nature of the disease and to improve the accuracy of the descriptions. The feedback from stakeholder interviews highlighted the importance of using qualitative methods with evidence generation [51].

In our study, NTDT-L and NTDT-H HSVs (which differ by Hb levels) were 0.78 and 0.63, respectively. The more burdensome transfusion-dependent HSVs (differing both in terms of transfusion burden and ICT) ranged from 0.75 for the mildest health state (LOSC) to 0.37 for the most severe (HSC). Our targeted literature review identified 31 studies that could inform the development of BT health states. Five of those studies addressed HRQoL aspects of BT health states in the UK. Among these, four did not elicit general population preferences, employ vignettes, or use TTO elicitation methodology, instead using 36-item Short Form (SF-36) questionnaire scores [43, 52–55].

One study by Karnon and colleagues used the TTO elicitation method with the UK general population [43]. It was, however, focused on the difference between ICT modalities and used only two vignettes, O-ICT or SC-ICT. The transfusion frequency used in Karnon et al. [43] was comparable to that implemented in this study for the medium burden health states (every 4 weeks). One other study that employed the TTO elicitation technique for the UK general population and used BT health state vignettes was accepted for publication after our literature review was conducted; it compared TDT health states (with either O-ICT or SC-ICT) with post-stem-cell transplant states [11]. However, no distinction was made between levels of transfusion burden. As the latter outcome was not of interest in our study, the only comparable values were the transfusion-dependent health states with differing ICTs. Matza et al. [11] report a HSV of 0.73 for a health state with O-ICT and 0.63 for a health state with SC-ICT. Karnon et al. [43], on the other hand, reported HSVs of 0.84 and 0.66, respectively. These values appear higher than the HSVs elicited in our study (0.60 for medium frequency with O-ICT and 0.54 for medium frequency with SC-ICT). HSVs in Matza et al. [11] and our study were observed to be lower than those in Karnon et al. [43]; these differences may be due to the higher proportion of university-educated respondents (closer to that of the general population, at 42% [50]) both in Matza et al. [11] and our study (43.5 and 48.9%, respectively) compared to 17% in Karnon et al. [43]. This aspect regarding potential education-related differences, together with age-related differences, should be considered in future TTO research.

Consistent with our findings, oral treatment was considered better than subcutaneous in both Matza et al. [11] and Karnon et al. [43]. It should be noted, however, that Matza et al. [11] and Karnon et al. [43] only considered a transfusion frequency of 3–4 weeks (equivalent to a medium/high transfusion burden in this study) and 4 weeks (equivalent to medium transfusion burden in this study), respectively.

HSVs derived in this study may be used in economic models that analyze and compare the value of treatments for BT. Importantly, all health states employed in this study were described as chronic and did not change over time. As such, the resulting utilities may be applied for any length of time in cost-utility models. We observed that participants more often disagreed on health states that were further away from full health, with higher variation observed for states with lower HSVs. This is consistent with the findings observed in the UK value set for EQ-5D-5L and EQ-5D-5L valuation literature [29, 56].

Current NICE guidelines for valuing health states refer to TTO approaches similar to the MVH protocol for conducting HRQoL preference elicitation studies [26]. Our cTTO study followed the EQ-VT protocol version 2.1 for the EQ-5D-5L instrument [57]. The EQ-VT and MVH protocols undertake the same approaches for the valuation of better-than-dead health states; however, the EQ-VT guidelines recommend the LT-TTO approach for worse-than-dead health states as a more accurate method of capturing preferences [31]. In line with the EQ-VT guidelines, we employed a QC procedure to assess data quality. We also utilized the reporting checklist compiled by Attema et al. [46] (co-authored by the EuroQol group), in reporting literature findings, methods, and outcomes. NICE Health Technology Assessment guidelines recognize the use of utilities derived from generic preference-based measures (such as EQ-5D) to promote generalizability and consistency [26]. This is particularly important when considering alternative studies attempting to measure patient QoL and elicit HSVs [23, 58]. Indeed, HSVs in two studies [23, 58] were generally higher than those identified by Matza et al. [11] and in our study. The inability of EQ-5D to accurately capture HRQoL in BT may well be the reason behind the use of SF-36 and the development of disease-specific instruments (i.e., the Transfusion-Dependent Quality of Life questionnaire [TranQol]) [26, 59].

Our study had a sample size of 200, which was deemed sufficient to achieve the stated objectives of this study based on the cTTO methodology protocol [44] and was in line with previous literature [11, 43]. However, the results should be considered in the context of some limitations. To reduce “respondent fatigue” and prevent data quality deterioration, the vignettes did not include all the possible health implications deriving from treatment or BT severity [60]. Additional limitations include protocol compliance and interviewer effects. Although every care was taken to train interviewers and ensure that they complied with the EQ-VT protocol [31], compliance issues were nonetheless recorded in some of the interviews and possible interviewer effects should be considered. One additional limitation is that, while participants were members of the UK general population and efforts were made (through recruitment criteria and targets) to ensure the sample was reasonably similar in terms of age and gender, it cannot be considered fully representative of the national population. Additionally, a degree of geographical bias cannot be ruled out, as only one geographic location was represented and interviews took place exclusively in the northwest of England, in a highly urbanized area (city of Manchester).

This study elicited utility values associated with BT from the perspective of the UK general population and, while efforts were made to provide essential information to participants, the vignette-based TTO approach cannot fully take into account all possible issues and complications related to BT and its treatment. Hence, the extent to which the utilities elicited in this study are fit for comparison with utilities derived from patients with BT is currently unknown. Additional research is warranted to assess patient valuations of these or similar health states, adding valuable information to the currently available body of evidence in this rare condition, as well as allowing for comparison between the patient valuations of health states and those presented as part of this and other studies eliciting utilities from the perspective of the general population. This may also be beneficial to research considering and assessing sensitivity of generic measures used to derive utilities in patient populations.

## Conclusions

This study examined HSVs across a range of NTDT and TDT health states from the UK general population perspective. In non-transfusion dependent health states, higher burden of anemia was found to be associated with lower HSVs. In transfusion-dependent health states, lower transfusion burden was associated with higher utility. Different ICT modalities may also contribute to QoL impact in BT, but to a lesser extent than treatment burden. The HSVs obtained in this study may be used in cost-effectiveness analyses of BT therapies. This work also highlights the unmet needs associated with treatment burden for patients with BT. The results observed in this study warrant additional research in BT with larger sample sizes and potentially involving participants with the disease to expand the body of literature around the burden and unmet need still characterizing this rare disease.

## Supplementary Information

Below is the link to the electronic supplementary material.

Supplementary file1 (PDF 1028 KB)

## Data Availability

Data requests may be submitted to Bristol-Myers Squibb Company, at https://vivli.org/ourmember/bristol-myers-squibb/ and must include a description of the research proposal.
