# Supplementary material for: Health state utilities for beta-thalassemia: a time trade-off study
Source: Eur J Health Econ. 2022 Mar 26;24(1):27–38. doi: 10.1007/s10198-022-01449-7 (PMC9876862; doi:10.1007/s10198-022-01449-7)

Electronic Supplementary Materials: ***European Journal of Health Economics***

Health state utilities for beta-thalassemia: a time trade-off study

Antony P. Martin[1], Enrico Ferri Grazzi[2], Claudia Mighiu[2], Manoj Chevli[3], Farrukh Shah[4], Louise Maher[3], Anum Shaikh[2], Aliah Sagar[1], Hayley Hubberstey[1], Bethany Franks[2], Juan M. Ramos-Goñi[5], Mark Oppe[5], Derek Tang[6]

[1]Formerly HCD Economics, Daresbury, UK; [2]HCD Economics, Daresbury, UK; [3]Celgene Ltd, a Bristol-Myers Squibb Company, Uxbridge, UK; [4]University College London Hospital, London, UK; [5]Formerly Axentiva Solutions, Tacoronte, Santa Cruz de Tenerife, Spain; [6]Bristol Myers Squibb, Princeton, NJ, USA

Corresponding author: Enrico Ferri Grazzi; email: enrico.ferrigrazzi@hcdeconomics.com

Appendix. Example of health states: HSC (A) and LSC (B)

HSC high transfusion burden with subcutaneous iron chelation therapy, *LSC* low transfusion burden with subcutaneous iron chelation therapy, *MRI* magnetic resonance imaging

A

<table>
<tr><th colspan="2">Part not visible to the respondent</th><th colspan="2">Part visible to the respondent</th></tr>
<tr><td rowspan="3">HSC</td><td rowspan="3">-Transfusion Dependent
-Iron Chelation Therapy: subcutaneous
-High transfusion burden</td><td>Blood Transfusion</td><td>• You receive blood transfusions every 3 weeks in the transfusion centre (hospital). Each transfusion takes at least 6 hours and most likely 9 hours in the transfusion centre (requiring 2 separate visits or sometimes being admitted overnight), requiring 1-2 days off work/education/family life. You are likely to have more than 4 transfusion visits in 12 weeks.
• During and after your transfusion (24-48 hours), you may experience <u>transfusion side effects</u> (fact sheet item 1).</td></tr>
<tr><td>Iron Removal Therapy: subcutaneous infusion</td><td>• You have regular iron removal treatment (5-7 days a week): <u>SUBCUTANEOUS</u> (fact sheet item 3).
• This treatment may cause some <u>side effects</u> (fact sheet item 3.1).
To monitor for potential the side effects, you require regular medical tests and MRI scans (requiring multiple hospital appointments each month).</td></tr>
<tr><td>Impact on your life</td><td>In general:
• You feel like you have little control over your condition.
• Your relationships and performance at work are affected.
• You may have low to moderate chronic pain.
• The high frequency of medical visits impacts work/education/family life.
After your transfusion:
• You may have some difficulties with daily life activities and may feel a little tired after normal tasks.
• Your performance at work may improve, but you may miss work for medical visits and blood tests.
• You have moderate disease-related stress. You can relax and enjoy everyday activities but are more nervous and restless.
For 7 days before your next transfusion:
• You may have difficulties with daily life activities and feel more tired than usual.
• You might experience: throbbing back pain, shortness of breath, fatigue, lowered exercise tolerance, palpitations, aching pain. You may have moderate-high chronic pain.
Your performance at work is affected (often too tired/sick to go to work).</td></tr>
</table>

B

<table>
<tr><th colspan="2">Part not visible to the respondent</th><th colspan="2">Part visible to the respondent</th></tr>
<tr><td rowspan="3">LSC</td><td rowspan="3">-Transfusion Dependent
-Iron Chelation Therapy: subcutaneous
-Low transfusion burden</td><td>Blood Transfusion</td><td>• You receive blood transfusions every 4-5 weeks in the transfusion centre (hospital). Each transfusion takes between 3-6 hours (and may require up to 8), requiring 1-2 days off work/education/family life. You are likely to have 2-3 transfusion visits in 12 weeks. During and after your transfusion (24-48 hours), you may experience <u>transfusion side effects</u> (fact sheet item 1).</td></tr>
<tr><td>Iron Removal Therapy: subcutaneous infusion</td><td>• You have regular iron removal treatment (3-4 days a week): <u>SUBCUTANEOUS</u> (fact sheet item 3).
• This treatment may cause some <u>side effects</u> (fact sheet item 3.1).
To monitor for potential side effects, you require regular medical tests and MRI scans (requiring multiple hospital appointments each month).</td></tr>
<tr><td>Impact on your life</td><td>In general:
• You feel like you have control over your condition.
• Your relationships and performance at work are slightly affected overall (you may be too sick/tired to go to work).
After your transfusion:
• You have no problems with daily life activities and no chronic pain.
• Your performance at work is not affected but you might miss work for medical visits.
• You have no disease-related stress. You can relax and enjoy everyday activities.
• You are fully able to participate in leisurely activities.
For 5-7 days before your next transfusion:
• You have no problems with daily life activities.
• You might experience: throbbing back pain, shortness of breath, fatigue, lowered exercise tolerance, palpitations, aching pain.
Your performance at work may be slightly affected (you may be too tired/sick to go to work).</td></tr>
</table>

Fig S1 Percentage of non-traders per interviewer.

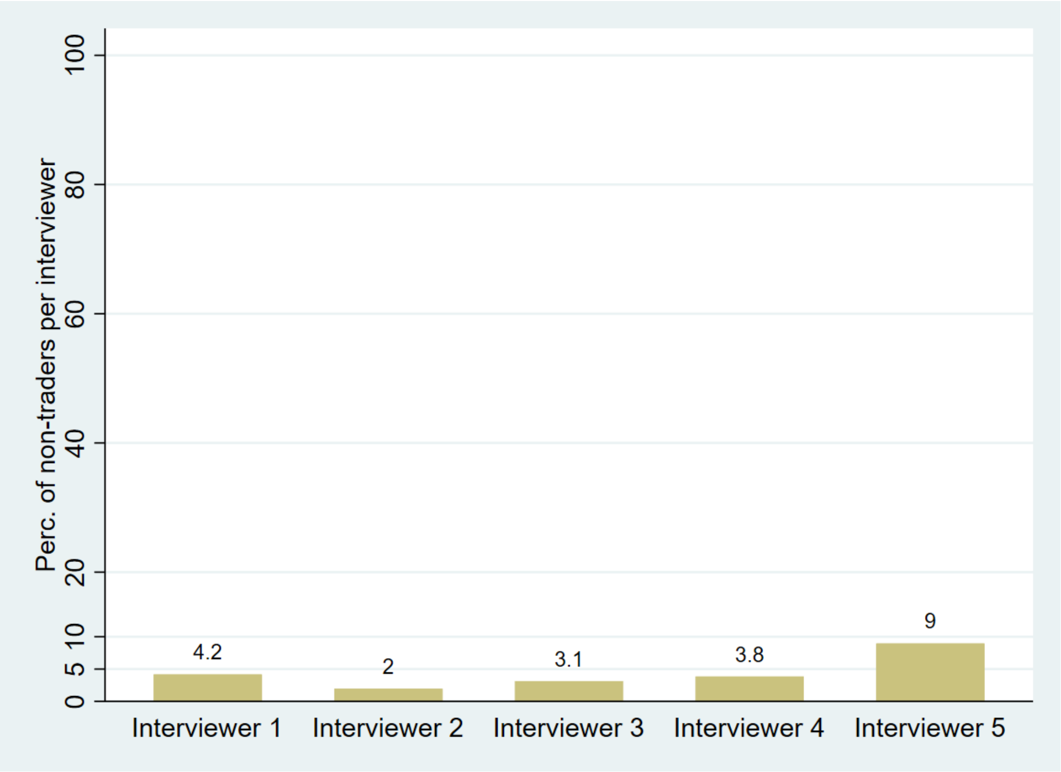

Fig S2 Density of zero-values per interviewer.

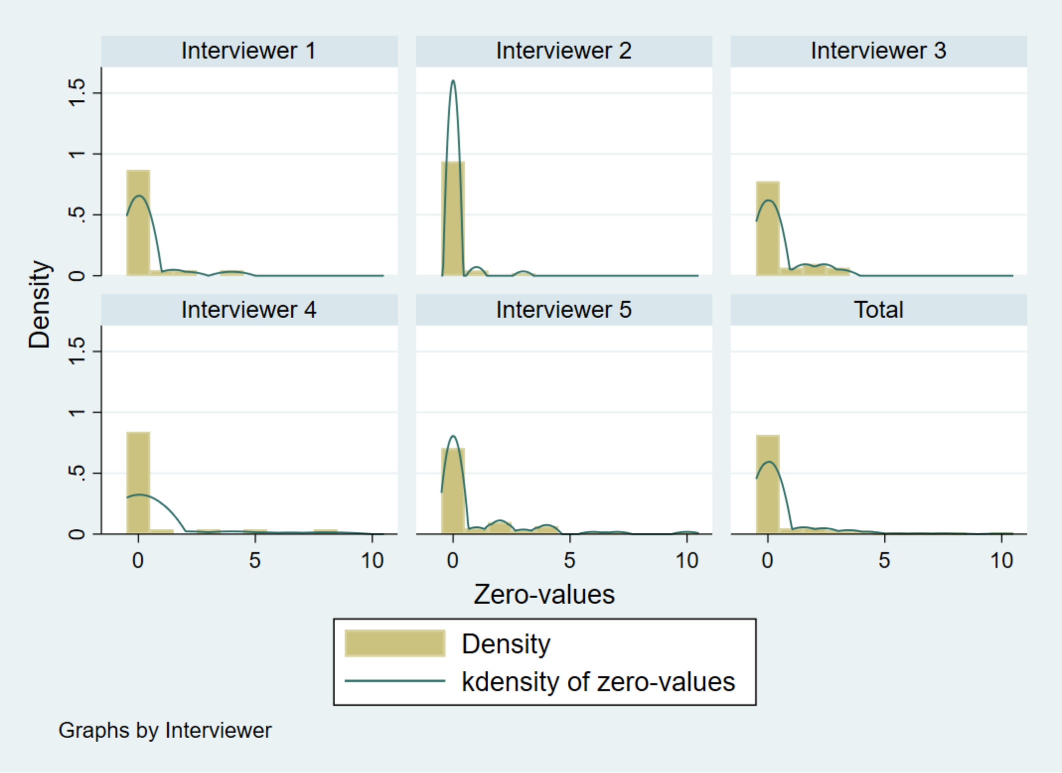

Fig S3 Percentage of WTD values per interviewer.
WTD worse than dead

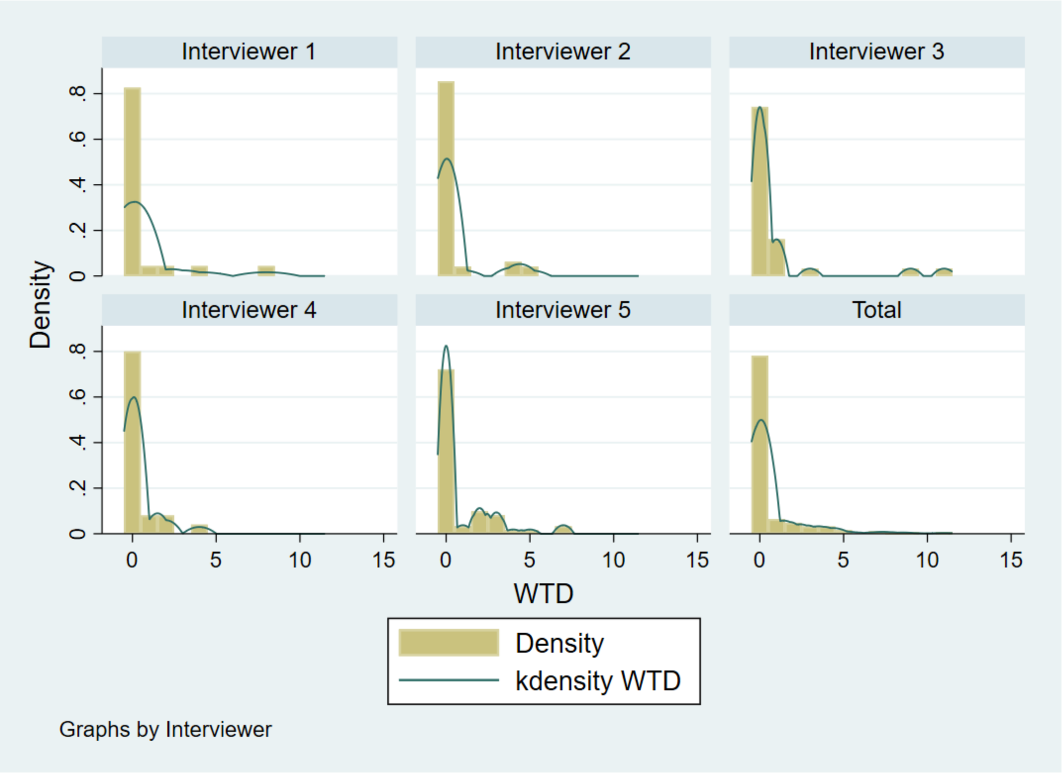

Fig S4 Density of number of moves per health state per interviewer

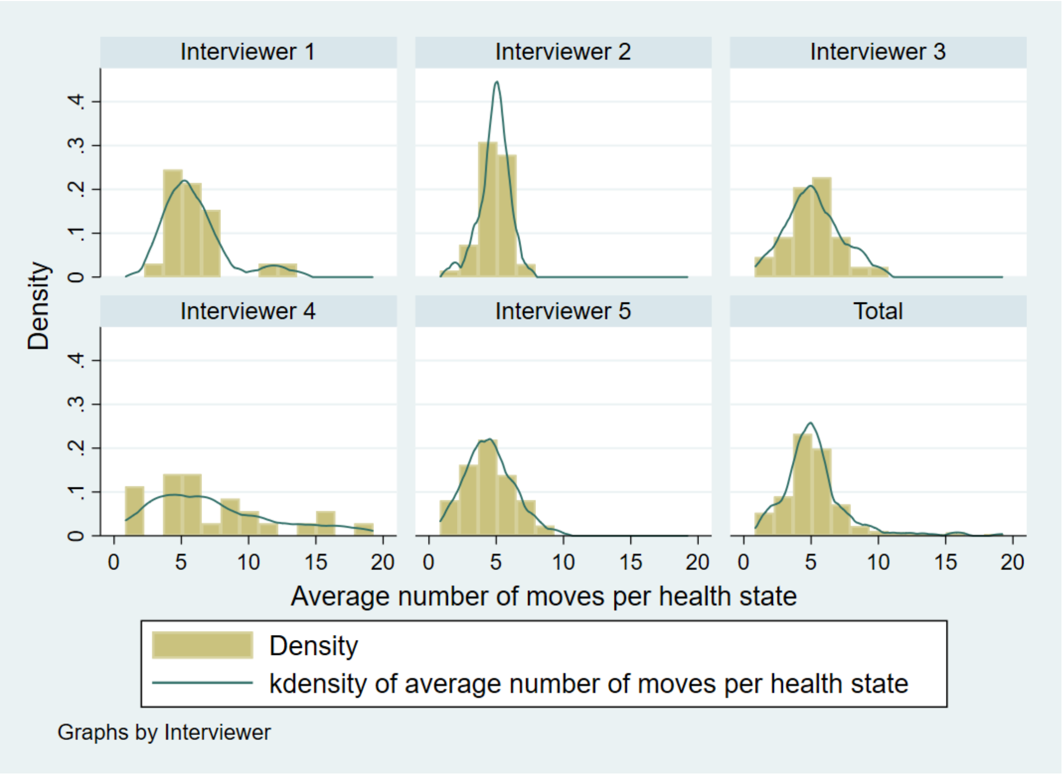

Fig S5 Average HSVs per interviewer
HSV health-state valuation

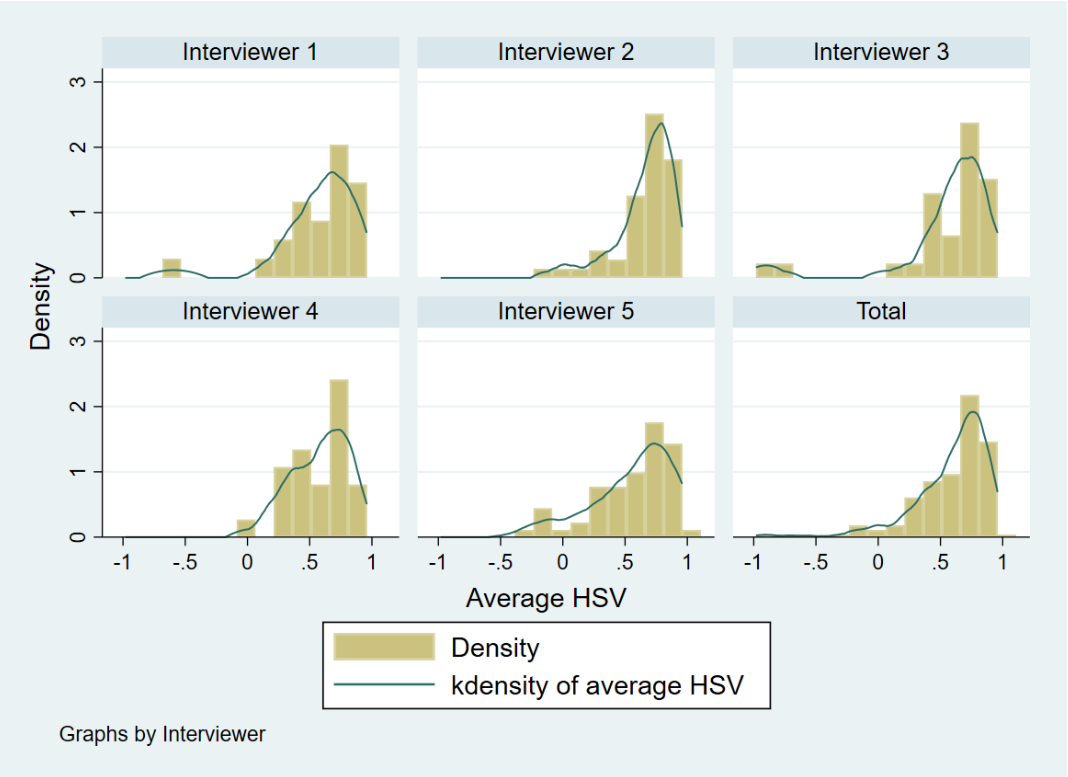

Fig S6 Face validity of data per health state

HO high burden (O-ICT), *HOSC* high burden (O- and SC-ICT), *HSC* high burden (SC-ICT), *ICT* iron chelation therapy, *LO* low burden (O-ICT), *LOSC* low burden (O- and SC-ICT), *LSC* low burden (SC-ICT), *MO* medium burden (O-ICT), *MOSC* medium burden (O- and SC-ICT), *MSC* medium burden (SC-ICT), *NTDT-L* non-transfusion dependent beta-thalassemia (low burden of anemia), *NTDT-H* non-transfusion dependent beta-thalassemia (high burden of anemia), *O-ICT* oral ICT, *SC-ICT* subcutaneous ICT

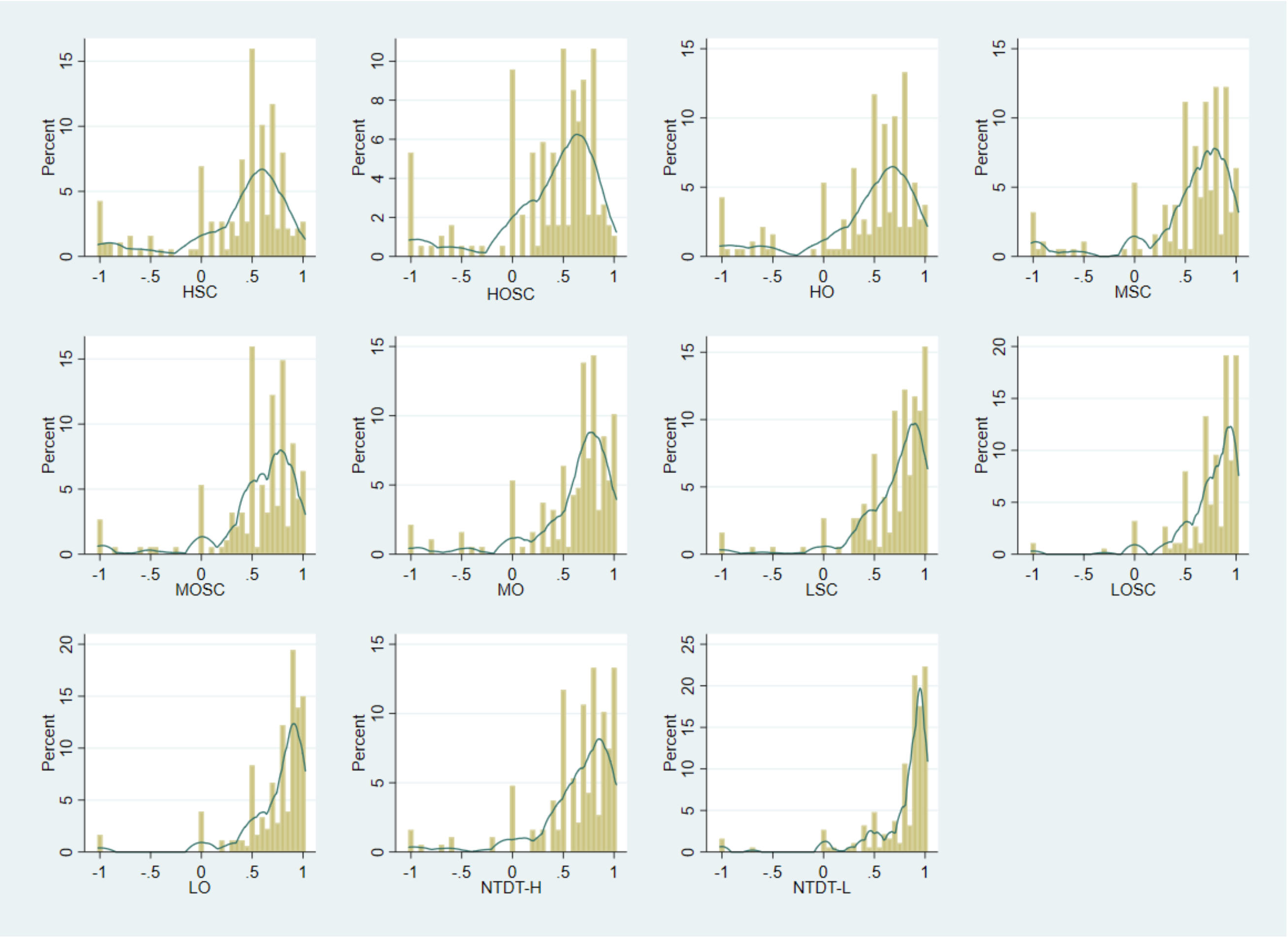

Fig S7 Face validity of HO data per interviewer.
HO high burden (O-ICT), *O-ICT* oral iron chelation therapy

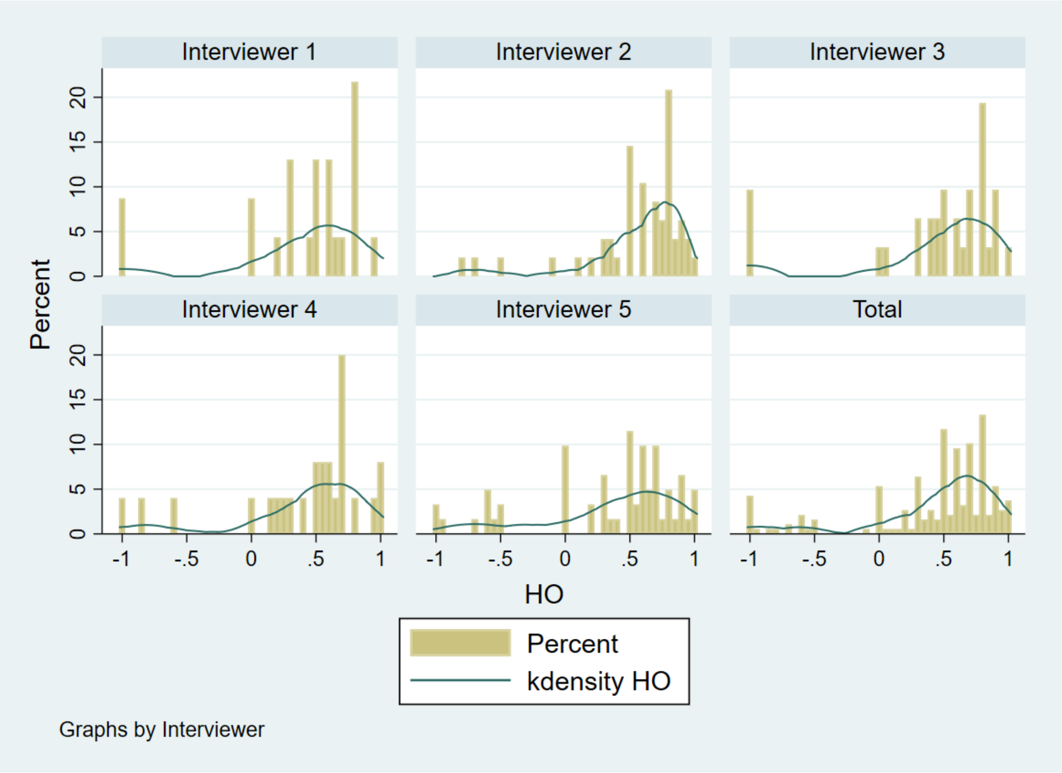

Fig S8 Face validity of HOSC data per interviewer
HOSC high burden (O- and SC-ICT), *ICT* iron chelation therapy, *O-ICT* oral ICT, *SC-ICT* subcutaneous ICT

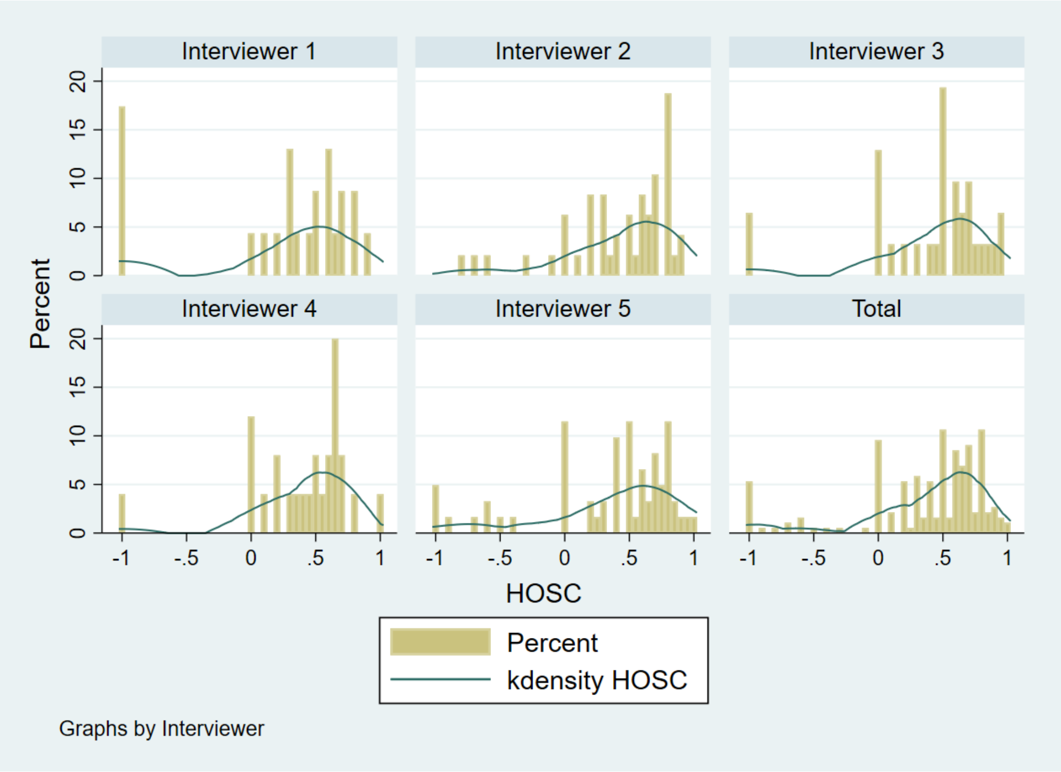

Fig S9 Face validity of HSC data per interviewer
HSC high burden (SC-ICT), *SC-ICT* subcutaneous iron chelation therapy

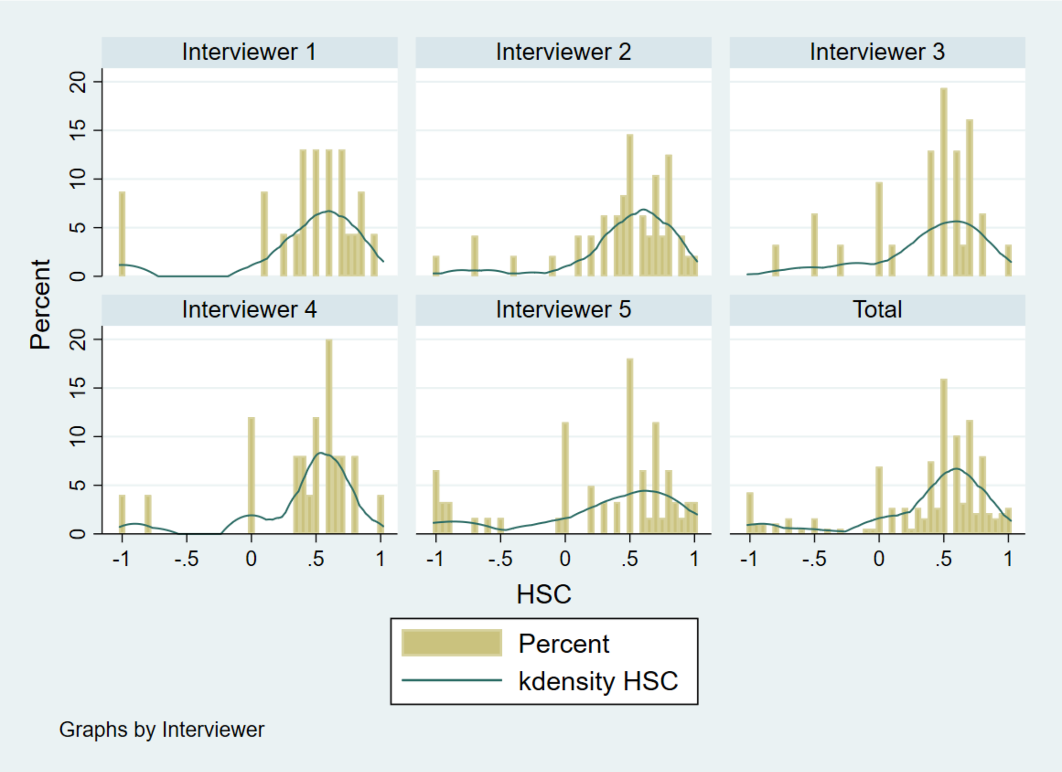

Fig S10 Face validity of MO data per interviewer
MO medium burden (O-ICT), *O-ICT* oral iron chelation therapy

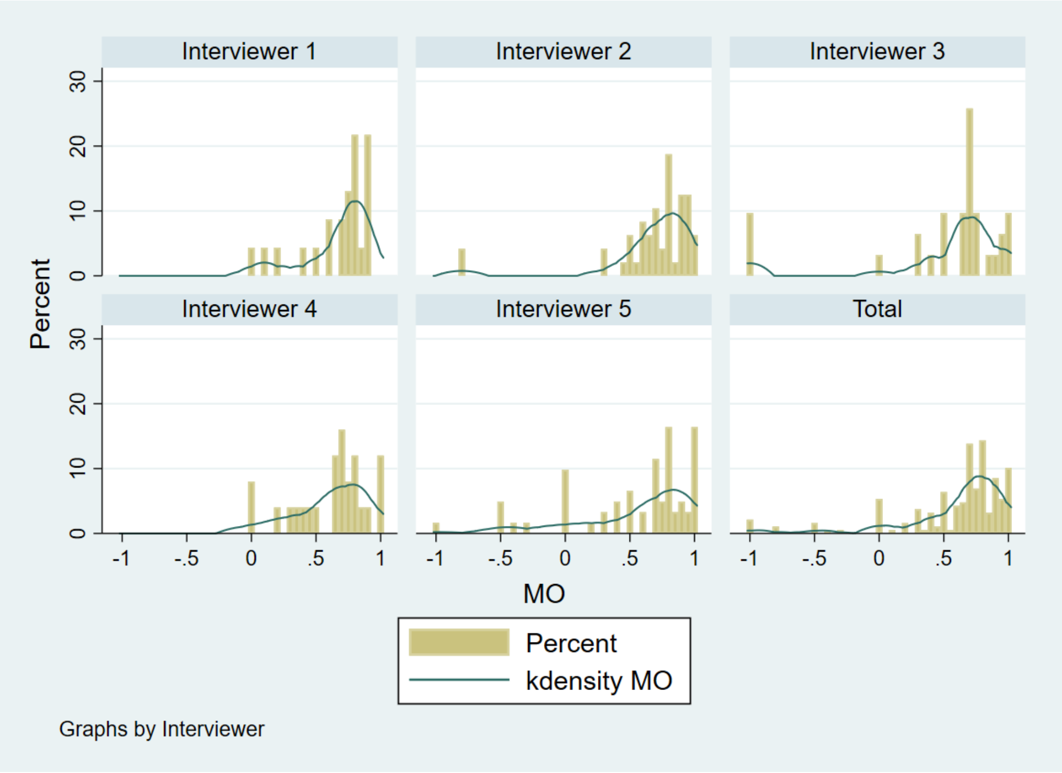

Fig S11 Face validity of MOSC data per interviewer
ICT iron chelation therapy, *MOSC* medium burden (oral and subcutaneous ICT)

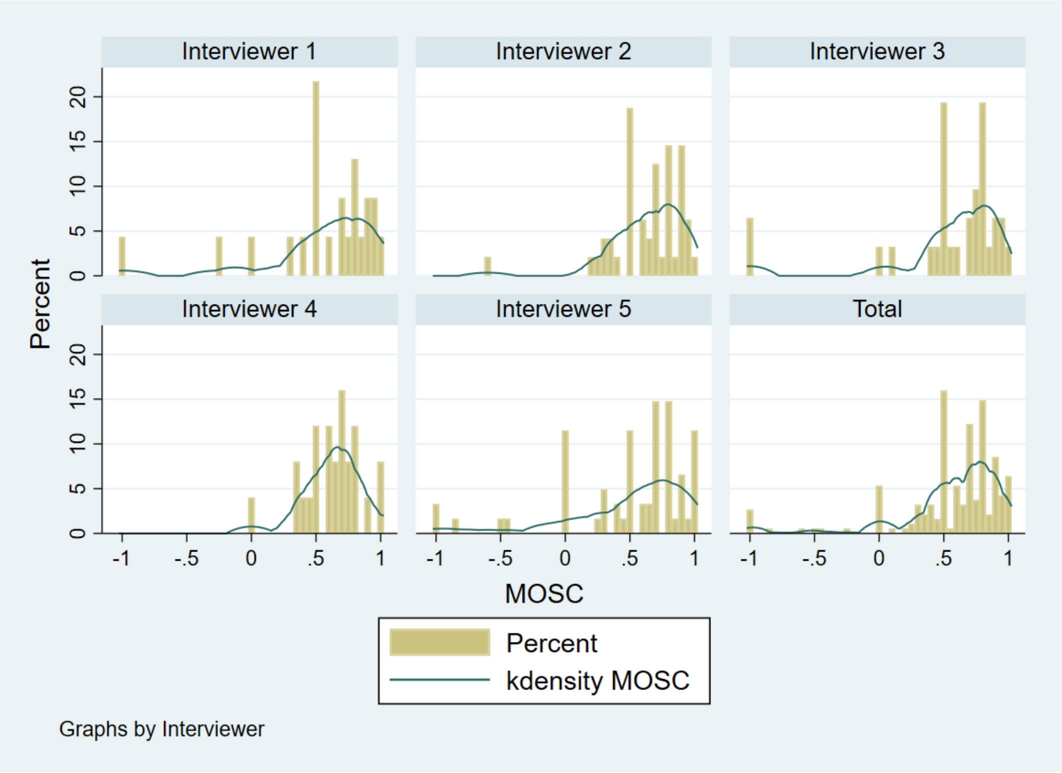

Fig S12 Face validity of MSC data per interviewer
ICT iron chelation therapy, *MSC* medium burden (O- and SC-ICT), *O* oral, *SC* subcutaneous

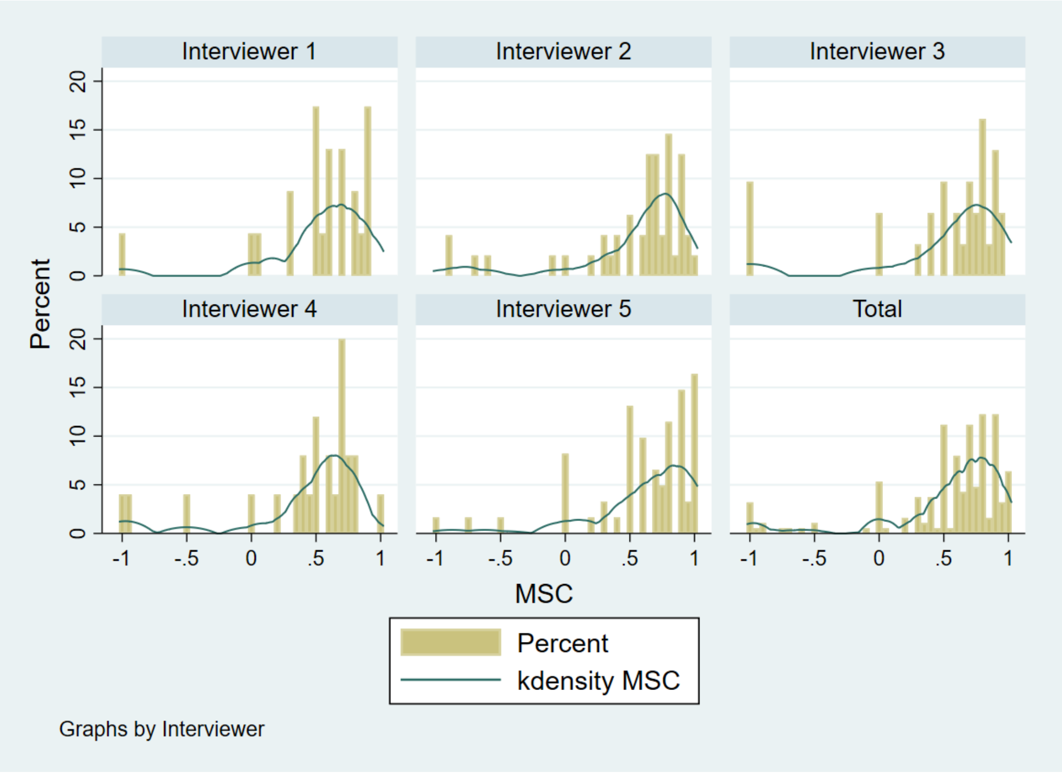

Fig S13 Face validity of LO data per interviewer
LO low burden (O-ICT), *O-ICT* oral iron chelation therapy

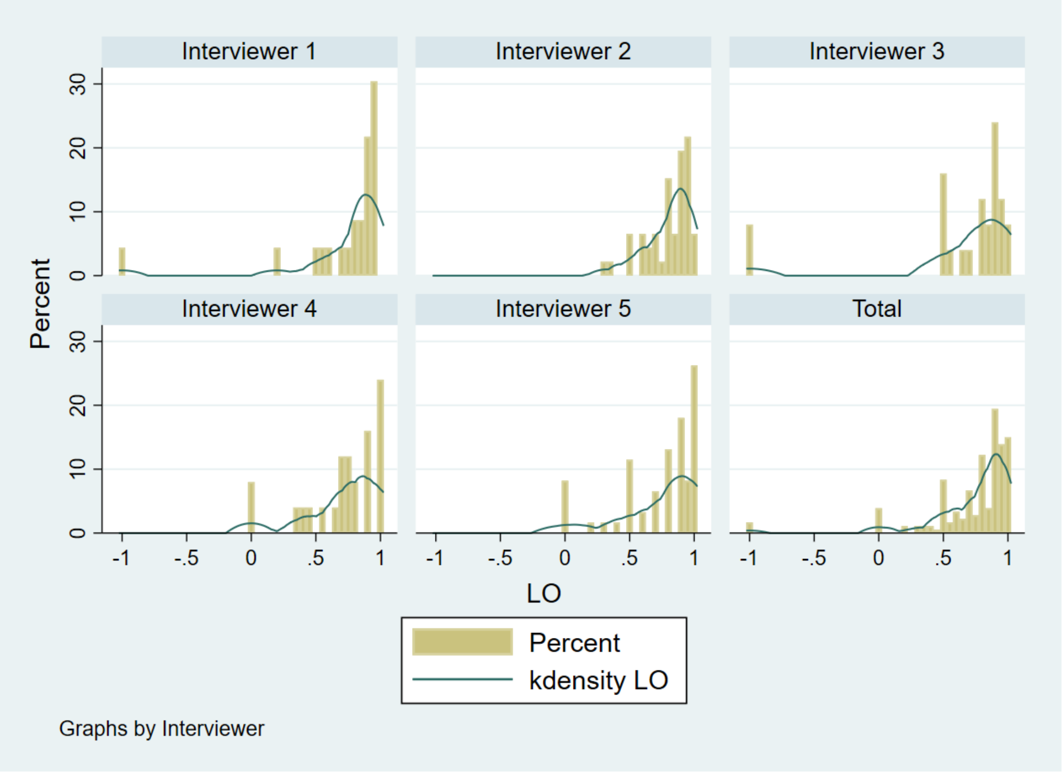

Fig S14 Face validity of LOSC data per interviewer
ICT iron chelation therapy, *LOSC* low burden (O- and SC-ICT), *O* oral, *SC* subcutaneous

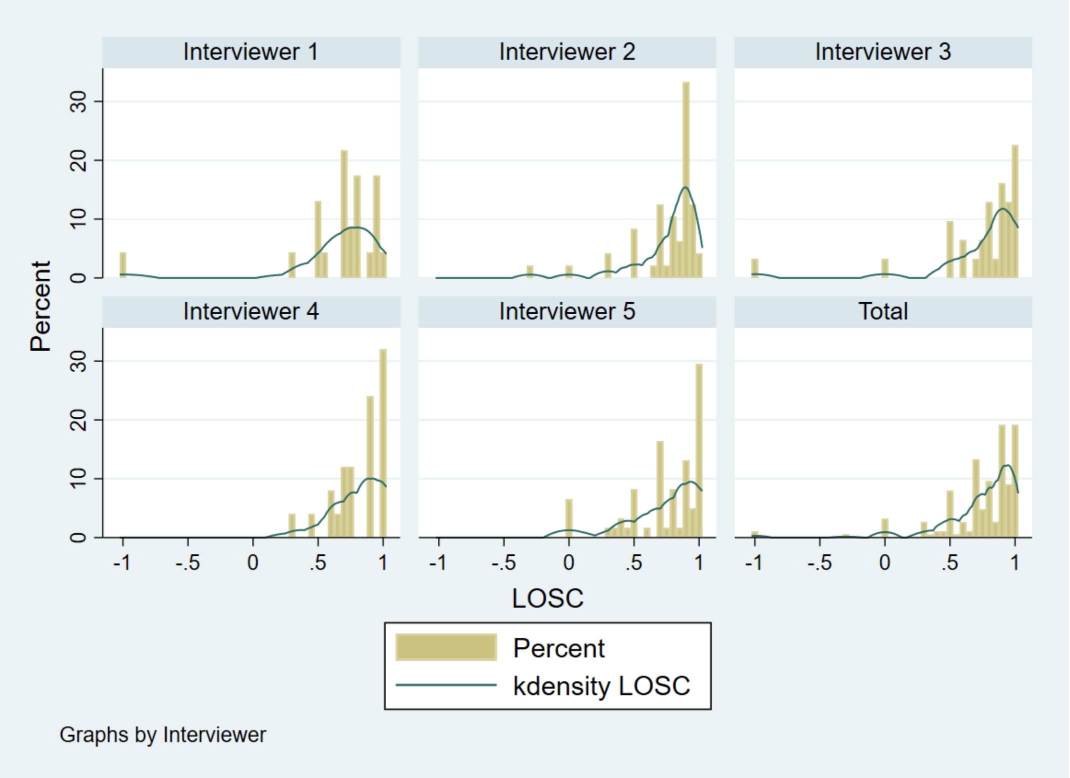

Fig S15 Face validity of LSC data per interviewer
LSC low burden (SC-ICT), *SC-ICT* subcutaneous iron chelation therapy

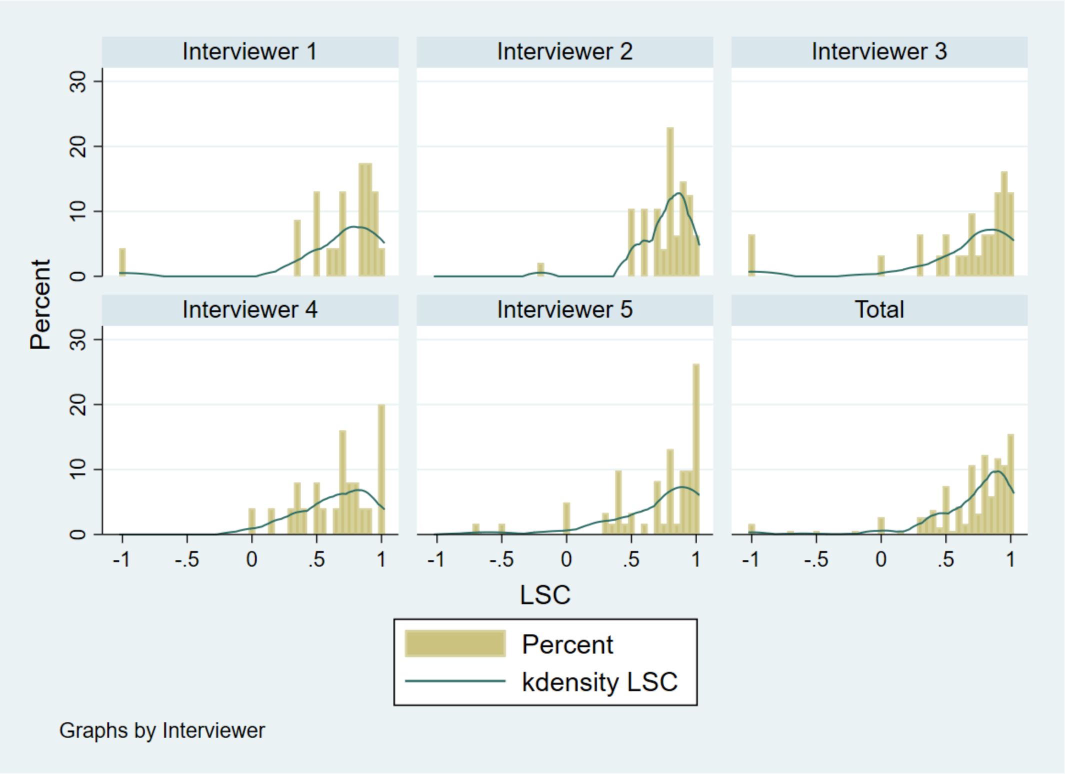

Fig S16 Face validity of NTDT-H data per interviewer
NTDT-H non-transfusion dependent beta-thalassemia (high burden of anemia)

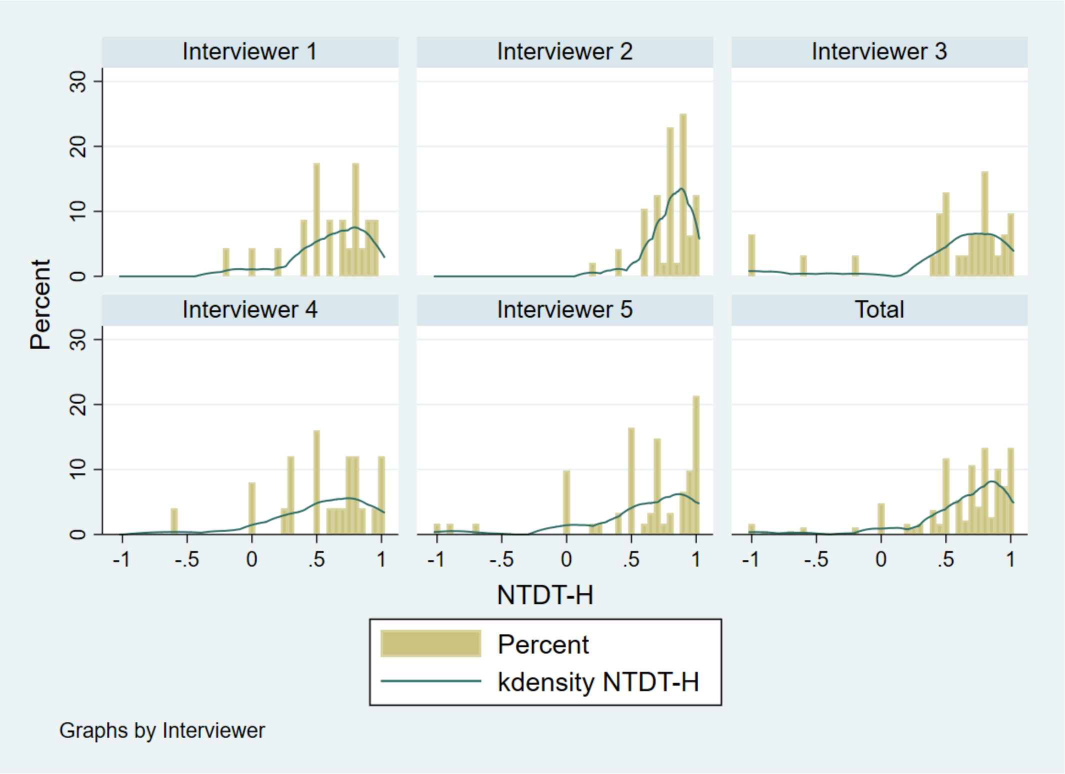

Fig S17 Face validity of NTDT-L data per interviewer
NTDT-L non-transfusion dependent beta-thalassemia (low burden of anemia)

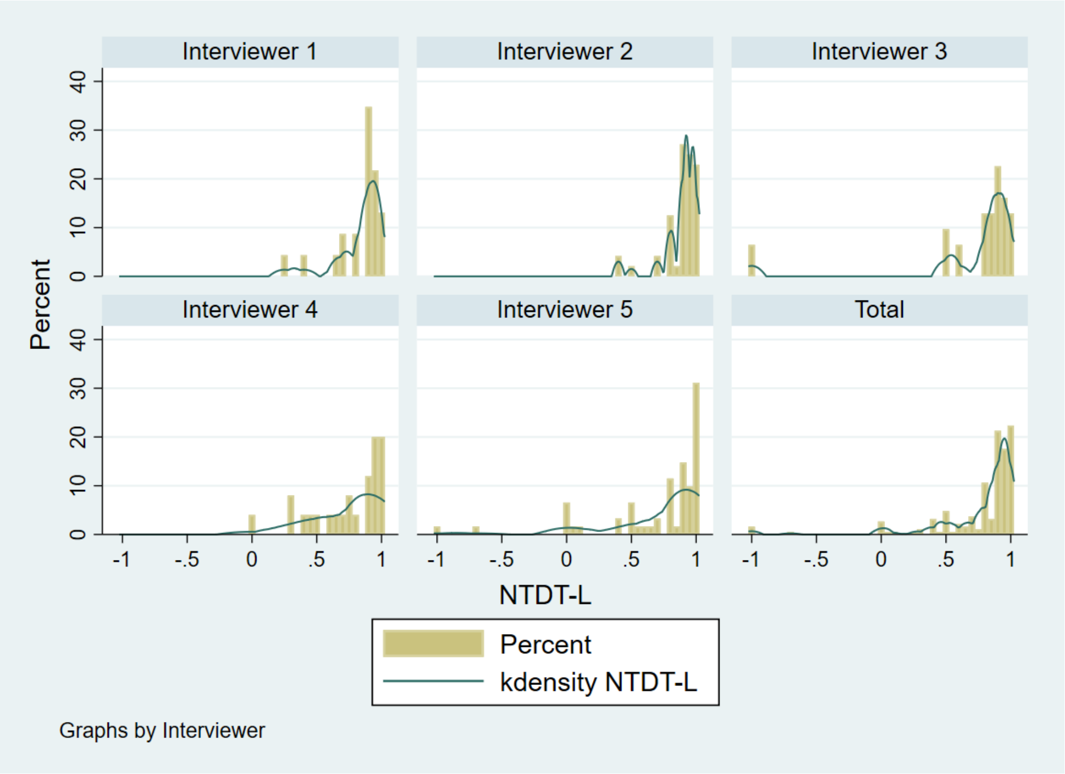

Supplement: Supplementary file 1 — Supplementary file1 (PDF 1028 KB) [file 10198_2022_1449_MOESM1_ESM.pdf]
